# Infiltrating history into the public health curriculum

**DOI:** 10.1093/pubmed/fdy058

**Published:** 2018-03-26

**Authors:** Virginia S Berridge

**Affiliations:** Centre for History in Public Health, London School of Hygiene and Tropical Medicine, 15-17 Tavistock Place, London, UK

**Keywords:** education, employment and skills, methods, public health

## Abstract

The insertion of history into the medical school curriculum has been discussed over a long period of time. But the role of history in the public health curriculum has not been the subject of much discussion, despite the changes in UK public health training and the advent of multidisciplinary public health. This article reviews the history of inserting history into the curriculum in a leading public health postgraduate institution. It discusses the strategies used to secure acceptance for history; the positioning of history within the curriculum both as a core and a special subject; and the different curriculum content and learning approaches which have been used over time. It reviews recent developments in distance learning and the launch of a history Massive Open On line Course. It concludes that no one approach can be recommended for inserting history and that flexibility, persistence, alliances and the willingness to adapt to local circumstances are important. Students themselves are now more receptive to historical approaches and can appreciate the value of a discipline which teaches critical skills of analysis and assessment of evidence. It remains to be discussed how the discipline and such approaches can be transferred into wider professional public health training and at the undergraduate level.

## Background

The purpose of this study is to examine the history of inserting history into the public health curriculum in a postgraduate public health institution and the lessons to be drawn for public health training. It is primarily a personal memoir, not a research paper. There has been wide discussion of the role of history in the medical school curriculum. But history in the public health curriculum has not received the same attention, although there has been much discussion of that curriculum in recent years.^1–3^ I will briefly review some relevant literature and the context of history and public health training. I will review strategies used to develop history teaching at the London School of Hygiene and Tropical Medicine; the structure of the programme over time; problems encountered; and ways forward.

## History in the medical and public health curriculum

The discussion of history in the medical school curriculum dates back to the 1940s and has continued.^4–6^ Some European countries, Germany in particular, have a long tradition of medical history teaching.^7,8^ The recent discussion in the UK dated from the publication of the General Medical Council’s 1993 document ‘Tomorrow’s Doctors. Outcomes and standards for undergraduate medical education’. History was one of several social science disciplines recommended with the potential to broaden the medical curriculum.The choice for medical schools was left open. Some hired sociologists, ethicists and anthropologists and others appointed historians. Wellcome Trust funding for research into the history of medicine and health was the wider context.^9^ A surge of interest occurred elsewhere: Jaclyn Duffin’s discussion of the insertion of history into the medical curriculum at Queen’s University (Kingston, Canada) was widely cited.^10^ Duffin was dealing with longer medical training and developed both core courses and electives. She stressed that there was no perfect format and that individual teachers and institutions had to develop a curriculum which suited local circumstances. ‘Tomorrow’s Doctors’, which went through several revisions, the latest in 2009, was replaced in 2016 by a new GMC document ‘Promoting excellence; standards for medical education and training’.^11^ The arrival of medical humanities more centrally has meant that the interest in history is less strong than it was. Nevertheless the legacy of the post 1993 change is notable, in particular in universities such as Liverpool and Birmingham which enthusiastically adopted the suggestions of the GMC document.^12,13^ Teaching is done by professional historians. However, a survey of history of medicine in Student Selected Components (SSCs) published in 2011 found that 15 of the 32 medical schools in the UK offered a history of medicine SSC. Most teaching was offered by medical professionals not by trained historians.The focus was on the 20th and 21st centuries.^14^

Public health has also undergone changes in its professional curriculum, reflecting changes in the workforce and the arrival of multidisciplinary public health. History plays almost no role in the Part A curriculum or the Faculty of Public Health training competencies. The competencies developed by ASPHER (Association of Schools of Public Health in Europe) do include history of public health and practice, alongside epidemiology, demography, sociology, social psychology and anthropology. A 2014 survey which mapped the core modules of public health Masters courses in the United States and in England showed no specific role for history in the core curriculum, with the exception of the Mailman School of Public Health at Columbia University, New York. There, public health history was part of a core course on the Foundations of Public Health, along with ethics and health and human rights (Document on public health training prepared for LSHTM teaching review 2014). There may be a history option available but it does not appear as a core requirement.

## Why history: when and what?

It is worth discussing briefly why students might gain benefit from studying history both of medicine and of public health; when they should do this; and what they should study.

The value of history lies, so it has been argued, in its ability to foster critical thinking and scepticism about the content of the formal curriculum. It can also foster critical analysis of historical ‘heroes’ (John Snow and Edwin Chadwick for public health). It inculcates the notion of change; that things have not always been as they are now and will change again in the future. History is recognized as a research discipline, with its own research questions, methods and materials, not so different from the research approaches of the basic sciences but with different questions, which can open up new issues for health professionals (Duffin, op cit, Sheard, op.cit). For those in policy positions, it also has relevance.^15,16^ When history should be studied has been discussed. Should it be a topic which is core for all students or should it be in a special study module available to those who particularly want to take it? Should history be a part of other topic based courses offered to students? What should the content of the history curriculum be? This is all discussed below.

## Infiltrating the curriculum

When I came to the London School of Hygiene and Tropical Medicine as Senior Lecturer in 1988, teaching the history of public health was initially far from my mind. I was part of a research programme, the AIDS Social History Programme, and, in those days, teaching was not part of the core requirement. Jane Lewis, Professor of Social Policy at LSE, had been taking history classes with Masters students at LSHTM after the publication of her book on the post war public health profession.^17^ She suggested collaboration to offer teaching on a wider basis, which I proposed to my new head of department. There had been history teaching in the School before. Major Greenwood, Professor of Epidemiology before World War Two, had given history lectures. Sidney Chave, a non-historian member of the Department of Community Medicine, had given lectures in the 1960s and 1970s, but these ended after his retirement. We have no knowledge of the content of Greenwood’s lectures. Chave’s appear, from his slides, to have covered a standard ‘heroic’ history of 19th century public health extending into topics such as health education and housing post World War Two.

Getting history into the curriculum was not easy. The School was going through extensive curriculum and other organizational change. For teaching, this meant central organization of the curriculum with core courses in term one followed by modular courses, some optional and some core, in terms two and three and a dissertation to complete the year. The changing public health curriculum was a battle ground for different interests: my development of history was disruptive of an agenda for the insertion of social science based on sociology. Misogyny (bullying, which led to the departure of one senior female member of staff) was also involved. An initial after hours history class offered to the students was deliberately disrupted by two colleagues sitting at the back of the class, talking and laughing. With persistence, a system for history emerged from these rough waters. I gained the support of the new Faculty teaching director, who wrote to all course organizers suggesting that they consider an historical lecture in their course. Several took this offer up. I lectured on the history of health promotion or of environmental health on core courses. A review of the history of the School was inserted into the introductory week. The core methods course, Principles of Social Research, took a lecture on the role of history and on historical methods. This integration into linear courses in term 1 prepared the way for a special history module, in term 3 after Easter. It had been agreed that it would be compulsory for students. But another course, also compulsory, was mysteriously timetabled at the same time. So I focussed my attention on getting it recognized by the organizers of different Masters in the School. A course which was ‘blobbed’ as a Masters option (given a blob against it for that Masters in the student handbook) was automatically higher profile with students. The strategy was one in which students overall were exposed to historical input in term 1 through a variety of entry points. Some chose to take the more specialist course in term 3. A pathway had been established. A competitive Wellcome funded Masters studentship for students choosing history gave some funding.

The content of the module has gone through three iterations. Initially I was the only historian on the School staff. I had experience in teaching non specialists through the University of London extra mural department and through my membership of the Open University course team on Health and Disease.^18^ The modular arrangement is a 5-week course in which the students spend half a week (2 and a half days) each week on the subject. I gave lectures on the history of public health overall and on health policy. I drew on the services of neighbouring London health historians such as Dorothy Porter on Europe. We visited the Wellcome library and archive and also the Science Museum. The module was assessed by presentation and essay and the marks formed part of the grade point average which went towards the final overall mark for the Masters.

Gradually the group of historians expanded. Jenny Stanton, who joined as research fellow in the mid 1990s, was a trained teacher. We tried a different approach. Rather than formal lectures, we asked the students all to read a core paper in advance of the scheduled teaching session. This would then be introduced and discussed with its implications drawn out. For example, we used Simon Szreter’s well known paper questioning the McKeown thesis and talked about its implications for both past and present.^19^ The assessed work was a research project of the student’s own choosing for which staff support was available. This produced some wonderful mini dissertations; for example, one on the history of the almost final smallpox outbreak in the UK, which had originated in the School. The student concerned tracked down papers and interviewed staff who had been there at the time (These projects have not been published although they may be still in LSHTM library).

The approach was too unstructured and thus difficult for some students. In ~2003–4 we developed the curriculum and approach which still operates, with some changes, today. The arrival of my historian colleague Martin Gorsky, another trained teacher, was very helpful. He imported techniques used with undergraduate history students at Wolverhampton University. The course starts with an exercise where students use and learn how to assess primary source materials to approach a big historical question. Lectures analyse the changes in what public health meant from the 18th century through to the near present. There are lectures on global public health; on sexual health; drugs and alcohol; the comparative development of health services; and bringing history into policy. A seminar follows,on a topic which expands on the lecture, for example, vaccination after the lecture on 19th century public health. The students have access (now on line) to seminar materials we have chosen, a mix of primary source materials and secondary historical analysis. These are read in advance and form the basis of a facilitated seminar discussion. The skills of assessing evidence and discussing historiography and interpretation are to the fore. Stand alone sessions cover public health films; a visit to the School archive, and to the Wellcome library. The assessed work is an essay based on primary and secondary source material, which we provide as part of the on line curated course material.

The positioning of the course had to change a few years ago. Bringing the School Masters curriculum in line with the European Bologna process meant teaching after Easter came to an end. We had attracted students who wanted to study a real interest after they had completed ‘bread and butter’ courses. Our internal module numbers, which had reached the upper twenties, dropped. But we reach a wide range of internal students in those terms now through the expansion of history lectures on topic based modules, including Social Epidemiology; Malaria; and Drugs Alcohol and Tobacco.

In 2011, colleagues and I published a book, ‘Public Health in History’, as part of the School’s distance learning (DL) series.^20^ There were difficulties getting history into the DL system because of the relatively small number of in-house students. Persistence on my part in forwarding the DL agenda paid off and the course has regularly attracted 50 or so students each year, covering similar ground to the in-house course. Assessment is by examination, although an essay may become the preferred option. DL is meant to be part of ‘blended learning’, where an in-house student can take a distance based course if there are timetable clashes.

A significant development has been a public health history Massive Open On line Course (MOOC). The School has developed very successful MOOCs on topics such as Ebola. The offer to develop a history MOOC came from the School, and my colleague Alex Mold took the lead. The course lasted 3 weeks in 2017 and was open to all. It covered post war British public health and attracted nearly three thousand students. It has run a second time and again attracted large numbers, with a third planned. Some internal students followed it.

## Future opportunities

Our in-house and DL courses regularly receive excellent evaluations and students ask why they are not exposed to more history earlier.A teaching review completed in 2014 did propose a Columbia style core course which would incorporate history for all,but the future of that initiative is uncertain. The expansion of historian numbers in the School—now three core funded historians and a range of research fellows—means that there is no problem in staffing. We have a tropical historian, John Manton, whose expertise fits well with the international students. The LSHTM History Centre has taken on the humanities brief and is currently developing ideas, such as our long interest in film and the media in teaching.

History teaching in the School is also delivered by non-historians. Auditing public health courses in the course of research for a book on public health, I discovered my non-historian colleagues gave lectures using history. Here the ‘old style’ history of public health was much to the fore and historical interpretation unknown. History is one of those disciplines which anyone thinks they can practise. Nevertheless these inputs also serve to acclimatize students to the idea of history and to give it legitimacy.

What we have developed in the School has not carried over into the wider area of public health professional training. The newer undergraduate degrees in Public Health do not incorporate history, at least from their published curricula. Returning to Duffin’s conclusions, it is indeed the case there is no one model which can be applied and no curriculum for all. Duffin’s model of core and electives has been achieved, although not in quite the same way. It has been important to be flexible, persistent and to keep long-term objectives in view. There has been a continual seeking of opportunities for development, in alliance with non-historian colleagues. Our students are not trained historians and have many demands on them in a 1-year Masters. Infiltrating the curriculum at various points has ensured that they have been given exposure to the critical analytical skills of history. It still remains to be acknowledged that these are also essential in the wider field of public health training.
